# Analysis of the influence of entrepreneurial psychology on the index system of digital development of the equipment manufacturing industry

**DOI:** 10.3389/fpsyg.2022.1026603

**Published:** 2022-11-08

**Authors:** Lu Zhang, Jian Li

**Affiliations:** ^1^School of Economics and Management, Yancheng Institute of Technology, Yancheng, Jiangsu, China; ^2^Business College, Yancheng Teachers University, Yancheng, Jiangsu, China

**Keywords:** psychology, entrepreneurship, manufacturing, digitization, indicator system

## Abstract

Entrepreneurs face the high pressure of the environment and may encounter psychological barriers such as entrepreneurial burnout. In order to maintain the growth of enterprises, entrepreneurs need to have toughness and perseverance to adapt to changes in the environment, overcome the negative effects of failure and crisis, and make progress in the crisis. The digital transformation of traditional manufacturing industry is an important way for the integration and development of digital economy and real economy. As the information support system of enterprise strategic psychology, strategic management accounting will also develop to a higher level with the digital transformation of enterprises. By reshaping an efficient spiritual organization, building an integrated innovation platform, and building an intelligent information architecture based on the concept of predictive finance, strategic management accounting will achieve important goals in the context of digital transformation. And give full play to the intellectual support role of its strategic psychology. The change of entrepreneurial psychology is crucial to the success of digital transformation of manufacturing industry. At present, the digital exploration of entrepreneurs’ psychological influence has made some preliminary achievements in technology and management, but according to the analysis in this paper, there is still much room for digital transformation.

## Introduction

Entrepreneurs create huge market wealth by promoting innovations in technology, management and business models, and drive business growth (Drejeris et al., 2021). However, environmental uncertainty causes enterprises to face survival crisis. In particular, the recent COVID-19 epidemic that has swept the world has not only seriously affected the survival and development of enterprises, but also caused serious damage to the psychology of entrepreneurs (Zada et al., 2021a). Entrepreneurs need the resilience and persistence to adapt to changing circumstances in order to sustain their business growth (Kim et al., 2020). Due to factors such as the low-end industrial structure, insufficient technological innovation, and declining demographic dividends, my country’s traditional manufacturing industries have begun to experience excess capacity and declining profits (Agbenyiga and Ahmedani, 2008). However, due to the difficulty of imitating and diffusing the core competitiveness of the manufacturing industry, and the increasing difficulty of my country’s manufacturing enterprises in learning from advanced countries, there are many obstacles to industrial transformation and upgrading (Aliaga-Isla and Huybrechts, 2018). Therefore, independent innovation has gradually become an important starting point for promoting industrial transformation and upgrading. As big data, cloud computing, artificial intelligence, electrification, networking, intelligence, and sharing are the development trends of the automotive industry, the rapid development of this trend is amazing (Ward et al., 1995). These “four modernizations” are all involved in the digital transformation of automobile manufacturing technology, enterprise organization, management and governance. Automobile is an important application carrier of technological revolution, and digital technology is more likely to usher in a subversive revolution in the automobile industry (Argyrou and Hummels, 2019). The networking of automobiles artificial intelligence, mobile Internet, Internet of Things, cloud computing, and big data, which will lead to qualitative changes in travel modes, traffic safety and efficiency, and energy consumption (Davidsson, 2015). Therefore, many countries and enterprises regard intelligent networked vehicles as an important development strategy (Zada et al., 2021b). China FAW is no exception, and its digital transformation has involved many aspects of R&D and management. The 21st century is the era of the transition from business management to entrepreneurial management.” Entrepreneurial activities inspire new thinking and promote new strategies, and at the same time help improve social and economic vitality and efficiency (Johnson and Mehta, n.d.). As the soul of entrepreneurial activities, entrepreneurial entrepreneurs are discussed in entrepreneurial management. The most topic. In the past 20 years, with the growth of small and medium-sized enterprises, a large number of studies on entrepreneurial entrepreneurs have emerged (Ford and Andersson, 2019). It is found that successful entrepreneurial entrepreneurs often have some unique psychological characteristics, try to analyze these characteristics and entrepreneurial success The relationship between entrepreneurship has become a hot topic in entrepreneurship research (Frolova et al., 2021).

Entrepreneurship is defined as risk-taking, predictable and violent product innovation activities.” The core of entrepreneurship is innovation (Zada et al., 2019). This paper focuses on analyzing the influencing factors of entrepreneurship from individual internal factors. Individual internal factors mainly include the entrepreneur’s personality, psychology and behavioral factors. The personal characteristics and psychological characteristics of entrepreneurs, such as success needs, risk preference, vitality, etc., affect the consequences of entrepreneurship (Andronikidis et al., 2021). Entrepreneurs play a decisive role in entrepreneurship: entrepreneurial tendency, adaptive adjustment ability, ability to obtain resources, etc. Psychological capital is the positive psychological state exhibited by an individual in the process of growth and development, and individual psychological factors that affect entrepreneurship, such as self-confidence, innovation, and taking risks are positive and developable psychological states, which are in line with psychological capital (Dohnalova et al., 2019). Standard. Research on the connotation of entrepreneurship based on the dimension of entrepreneurial psychological traits (Prado Maillard et al., 2019). There are two main aspects of foreign research on entrepreneurship. “Witty, firm, prudent and enterprising.” The sample of this paper is selected from a sample survey of 150 people, so as to obtain data and carry out the next calculation and investigation. On this basis, further research came to the view that entrepreneurs have subjective initiative in uncertain circumstances, and entrepreneurship is the quality of daring to take risks, to create and to take risks Second, among many viewpoints, one of them stands out. The unique means of entrepreneurs is innovation, and entrepreneurship is an action rather than a temperament (Zada et al., 2022). Opportunities to take full advantage of. Entrepreneurship can be defined from three aspects: innovation, active competitive attitude, and risk-taking.

## Entrepreneur’s psychological influence

### The impact of entrepreneurial psychology on enterprise risk

Entrepreneurship is the core concept of entrepreneurial research at home and abroad. It can not only be translated into “entrepreneurial spirit” and “entrepreneurial ability,” but also includes the connotation of entrepreneurship and innovation. It is a series of behaviors characterized by discovering opportunities and taking risks. Process (Marie, 2016). Entrepreneurs are the main actors in it. In recent years, with the development of human capital theory, there have been more and more discussions on the exploration of entrepreneurial issues based on the type of human capital and even the dynamic development characteristics of human capital (Marie, 2016). Combining these discussions, we can roughly base on the reality of enterprises and entrepreneurs are divided into two types: business-oriented entrepreneurs and entrepreneurial-oriented entrepreneurs (Glaeser and Xiong, 2017).

### Entrepreneurial responsibility and cooperative awareness

Responsibility awareness is a positive contract spirit, which is manifested in the entrepreneur’s courage to shoulder heavy burdens and take responsibility in corporate activities. Including economic responsibility awareness, legal responsibility awareness and social responsibility awareness (Beland et al., 2020). The board of directors is responsible for the company’s strategic psychology and the supervision of the management’s implementation of the strategic psychology, and is the company’s core governance body. Implement online general meeting of shareholders to encourage more investors to participate in the company’s psychology and supervision. The economic responsibility awareness index selects the return on net assets and the asset-liability ratio, which reflects the responsibility of the enterprise to the stakeholders in the economic activities; the legal responsibility is the responsibility that should be assumed by the laws of our country, and the index selects the *per capita* salary of employees and the tax rate of assets; Social responsibility is the return of entrepreneurs using their own value to the society, and the indicators are selected employment contribution rate and donation income ratio, reflecting the ability to solve employment problems and charitable performance. Risk-taking consciousness is the entrepreneur’s will to take risks and forge ahead in order to achieve their goals. In the face of complex information and many uncertainties from inside and outside the enterprise, entrepreneurs need firm will and the ability to practice (Plaskoff, 2012).

## Manufacturing digital transformation

### The development of manufacturing informatization

The “digital economy” first appeared in the 1990s. In 1998, a research report by the US Department of Commerce described the development trend from an industrial economy to a digital economy driven by the diffusion and penetration of IT technology (Oztemel and Gursev, 2020). The digital economy is defined as a special economic form, pointing out that the essence of the activities of the digital economy is “the transaction of goods and services in the form of information.” The digital economy includes the information and communication technology (ICT) industry, the information technology industry, and the retailing of goods and e-commerce based on the information technology industry (Ghobakhloo, 2020). The core of the digital economy lies in the information and communication technology (ICT) technology, and the promotion of ICT technology to traditional industries is the future development direction of the digital economy. The G20 summit held in Hangzhou in 2016 further clarified the definition of the digital economy (Ivanov et al., 2019): Taking digital knowledge and key production factors, taking modern information network as an important carrier, and taking effective utilization of information and communication technology as high efficiency and economic development. An important driving force for the optimization of a series of economic activities. It can be seen that the essence of the digital economy is an economy, which is a higher economic stage after the agricultural economy and the industrial economy. The core connotation of digital lies in the application and empowerment of information technology in entities, which is a deep integration of the two.

### Optimal configuration of digital economy

The digital economy is an economic form that guides and realizes the rapid optimal allocation and regeneration of resources through the identification, selection, filtering, storage and use of big data technology, thereby achieving high-quality economic development. At the technical level, it covers emerging technologies such as big data, cloud computing, Internet of Things, blockchain, artificial intelligence, and 5G communication; at the application level, it includes typical representatives such as “new retail” and “new manufacturing.” In recent years, my country’s digital economy has developed rapidly (Ivanov et al., 2019). According to the “Digital China Construction and Development Report,” the scale of China’s digital economy reached 31.3 trillion yuan in 2018, and it will account for more than 50% of GDP by 2030. To promote the transformation of enterprises and society, and to promote the transformation of industrial development mode. The application of digital economy has attracted widespread attention from scholars (Zhou et al., 2018). In the industrial transition, the digital economy through the allocation, penetration and integration, and industrial synergy has greatly improved the prime productivity and promoted the structural adjustment of the manufacturing industry. In the digital age, on the one hand, the integration of Internet manufacturing has provided a huge impetus for manufacturing innovation, product innovation, model innovation, business innovation, management innovation, etc.; The resource method and organizational process are accelerated from being producer-centered to consumer-centered, forcing traditional enterprise fields to accelerate the construction of innovation systems.

## Psychology and transformation factors research model

### Conception and assumption

**Figure 1:**
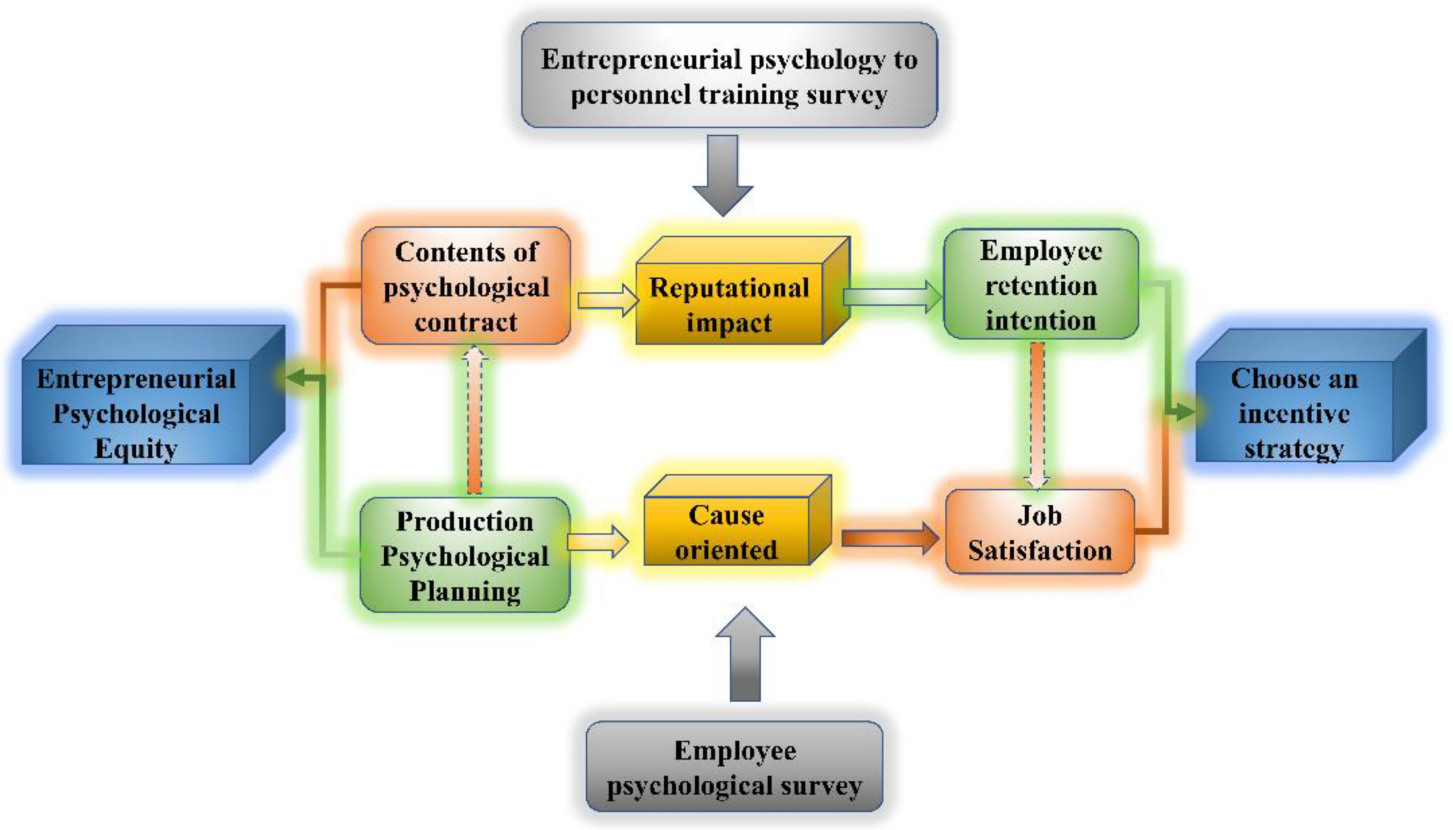
The extraction process of manufacturing transformation mental organization types.

On the basis of clarifying the research topic, the literature related to organizational inertia is first reviewed to determine the definitions of the concepts and types of organizational inertia in existing research, and to clarify the manifestations and manifestations of different types of organizational inertia. Inertia. preliminary data. Through typical representative research, the definitions of organizational inertia concepts such as cultural inertia, behavioral inertia, technological inertia, strategic inertia, structural inertia, and economic inertia are refined and sorted out (Trantopoulos et al., 2017). In this way, the types of organizational inertia that do not exist in the data are eliminated, and the identified organizational inertia and the transformation stage in the process of digital transformation are coded and analyzed. And represent the stage codes of online retail, platform, and ecology, respectively. Figure 1 shows the extraction process of psychological organization types and manifestations in the process of digital transformation of traditional manufacturing enterprises. This paper adopts the grounded theory method to continuously deduce and summarize the identified organizational inertia in digital transformation until the organizational inertia theory in different stages of digital transformation is saturated.

### Research hypothesis

Psychological contract violation will have a significant impact on the entrepreneur’s work behavior and attitude. If the psychological contract is violated, the entrepreneur’s satisfaction and desire to stay will be reduced. The entrepreneur’s sense of organizational justice is another important factor that directly affects their work attitude. Psychological violations of different dimensions in the entrepreneur’s sense of organizational fairness have different influences on it. Entrepreneurs’ job satisfaction is closely related to the attribution of their psychological contract. This paper adopts the two-dimensional method of digital technology application and business model innovation to measure the digital transformation of enterprises. Specifically, when extracting keywords, this paper firstly uses a third-party natural language processing library, to segment Chinese texts. By calculating the TFHDF (term frequency—inverse document frequency) value of each word in the root, the text keywords are analyzed, and the first five words in each text are selected as candidate keywords. Secondly, based on the existing research experience and the text characteristics of the enterprise digital domain, the method of manual extraction is adopted. After analysis and confirmation, 89 domain keywords are finally obtained from 125 candidate keywords. Third, on the basis of determining the basic keyword database, combined with the context and semantic context analysis, the method of learning is used to further determine the synonyms of the words contained in the basic keyword database by calculating the similarity. Word vectors, then people. The screening method determines the final keyword library. Finally, this paper captures relevant sentences in the annual report containing Guanku, and judges the relationship between the captured sentences and the digital transformation of enterprises through manual reading. Business model innovation is reserved. If the captured statement indicates that the company may innovate in digital technology and business model in the future, it will be excluded. Entrepreneur’s behavioral choice and the degree of emphasis on reputation are closely related to entrepreneur’s psychological contract violation. Through the paired sample *T*-test method, the data collected from the entrepreneur’s psychological contract scale is compared with the psychological contract violation scale.

### Research sample test

After the questionnaire was collected, data sorting and statistical analysis were carried out, and item analysis was carried out for each item that violated the psychological contract. Explanatory variable: Whether the enterprise has undergone digital transformation (Digit) o If the enterprise has undergone digital transformation in the current year, take 1, otherwise it is 0. Explained variable: Audit quality (DACC). High-quality audit can effectively restrain management’s opportunistic behavior and improve the company’s earnings quality. Therefore, in the main regression, this paper chooses accrual earnings management as the proxy index v of audit quality and selects the modified Jones model to measure manipulative earnings. In order to more comprehensively examine the impact of digital transformation of enterprises on audit quality, this paper refers to the existing research “, selects the domestic “top ten” manufacturing enterprises as the surrogate variable for high audit quality in the robustness test, and selects the logarithm The post-audit fee is used as a surrogate variable for audit quality.

**Figure 2:**
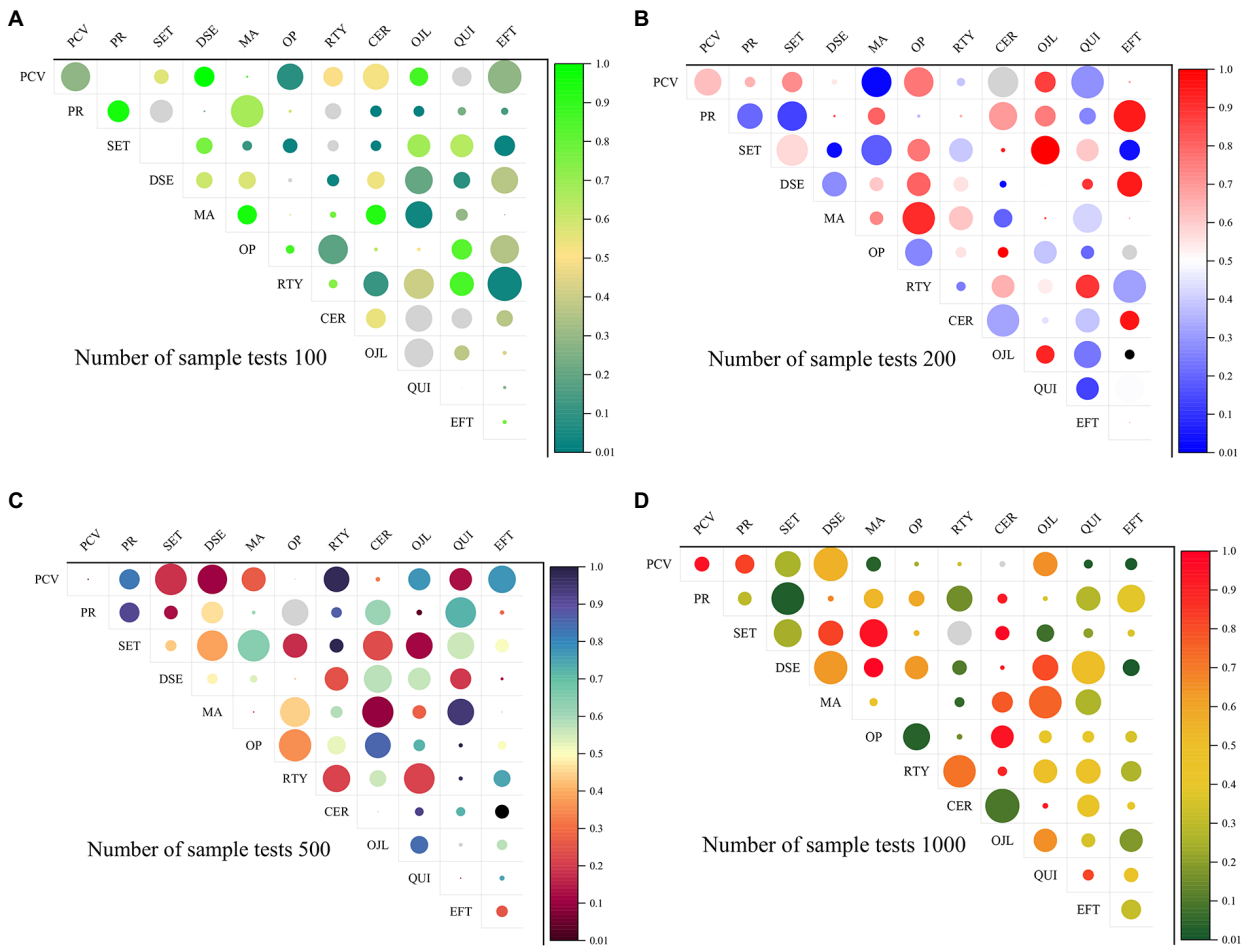
T-test results of each psychological index of the sample. **(A)** Number of sample tests 100. **(B)** Number of sample tests 200. **(C)** Number of sample tests 500. **(D)** Number of sample tests 1,000.

**Figure 3:**
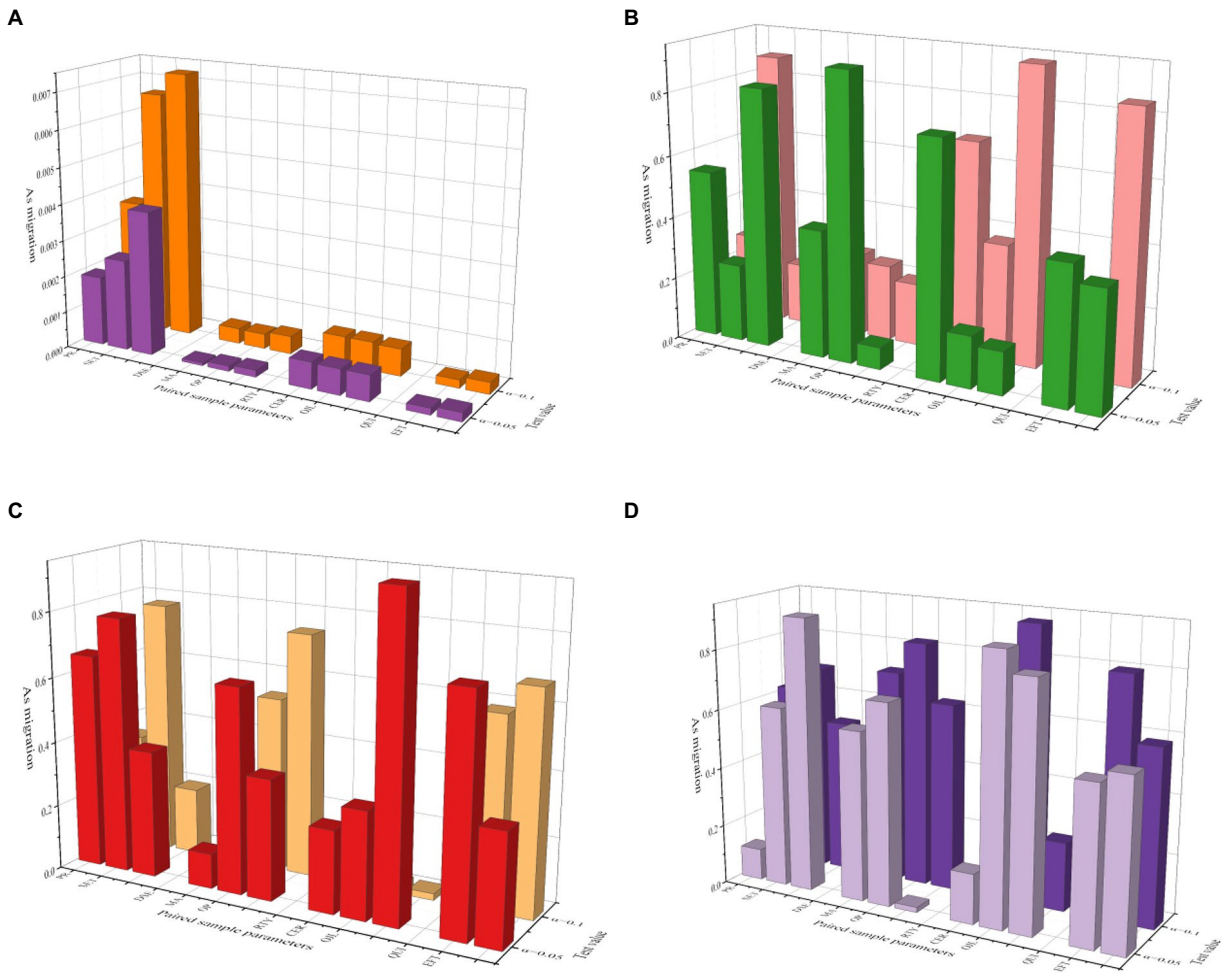
Trend of AS transfer rate.

The specific method is to calculate the total score obtained by the subjects in the scale, the first 27% of the scores are classified as high group, and the last 27% of the score is classified as low group, and each question is subjected to an independent *T*-test to detect the average number of each question. If the CR value of the item reaches a significant level of 0.05, it means that this item can identify the degree of response of different subjects, and the item will be retained; otherwise, if it does not reach a significant level of 0.05, it will be considered whether to delete the item. The problem. The CR value of each item has reached a significant level, indicating that the questionnaire has acceptable reliability and does not need to be deleted. Figure 2 is a paired comparison of the collected data, and the paired sample *T*-test results. It can be seen in Figure 2 that with the increase of the number of samples from 100 to 200, the heterogeneity is significantly reduced, which shows that the analytical model is relatively stable in a small number of samples. However, when the number of sample experiments increased from 200 to 500, the heterogeneity decreased, and the numerical distribution of sample model analysis was more uniform. When the number increases from 500 to 1,000, the homogeneity of the entire data distribution decreases significantly. It can be seen that this model is suitable for the number of samples around 500, and the data in this paper is true and effective. Figure 3 is a trend diagram of the AS transmission rate under different test values and confidence intervals, where the confidence levels from Figures 3A–D are 0.01, 0.02, 0.05, and 0.1. In the process of analyzing the data graph in Figure 3, we found that when the confidence level was 0.01, the whole model played poorly, the data were small, and did not reflect uniformity and dispersion, so the data credibility was poor. When the confidence level is 0.02 and 0.05, the whole sample distribution is relatively uniform, which can reflect the superiority of the model over other confidence levels. When the confidence level is 0.1, large data and small data have a large degree of dispersion, and the whole data is distorted, losing the reliability. Therefore, the confidence level of this model should be more accurate between 0.02–0.05.

### Entrepreneurial psychology and employee turnover

**Figure 4:**
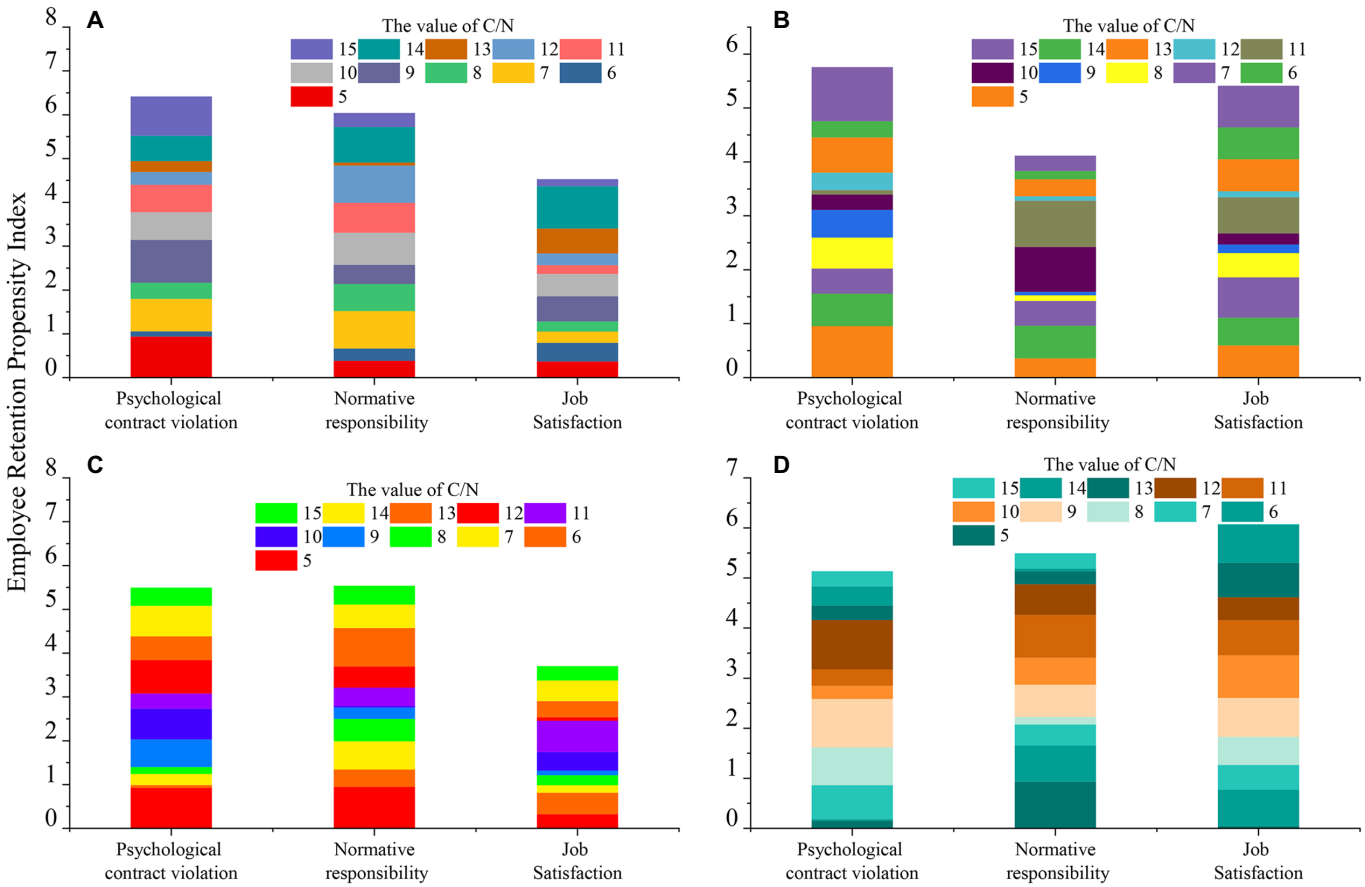
Manufacturing psychological factors and employee retention impact.

The underlying technology architecture of enterprise digital transformation, including artificial intelligence, blockchain, and big data technologies; “Technology Practice” focuses more on the specific application of digitalization in different business scenarios. The number of keywords is added by total (DCG) and category (KDT, ADT). After logarithmic processing, regress. We processed the survey data using SPSS 13.0 statistical software and performed sub-analysis (or factor analysis) and internal one-digit analysis to test the conceptual model and associated hypotheses of this study. We used the methods of correlation analysis, regression analysis and one-way analysis of variance. In the process of studying the relationship between normative responsibility, psychological contract violation and employee satisfaction to retention willingness. The method of C/N index correlation analysis is adopted, and the results are shown in Figure 4. Figures 4A–D correspond to the operation time of 5, 10, 15, and 20 s, respectively. In the analysis of Figure 4, when the model analysis time is 5 and 10s, the top squares of Figures 4A,B account for too much and have poor uniformity. With the increase of time, when the time is 15 and 20s, it is obvious that the grid size of each part of the stacked graph is relatively uniform, and the data error is small. As the time for model analysis increases, the data uniformity is getting better and better, and the error is getting smaller and smaller. In the practical application of the model, it is necessary to try to increase the operation time of the model, so as to improve the accuracy of the operation.

## Future development direction

Through empirical analysis, we can see that under the influence of the current network environment, entrepreneurs’ psychology has a certain impact on enterprises’ innovation behavior. From the perspective of correlation analysis, entrepreneurial psychology has established an investor (shareholder) relationship management network, which has unblocked the information channels between investors and between investors and companies. A good investor relationship is an important prerequisite for the stable development of enterprises. After studying the influence of entrepreneur psychology on digitalization of manufacturing industry, entrepreneurs should improve their own quality and attach importance to psychological capital investment in the process of digitalization transformation of enterprises in the future. Entrepreneurs should not only rely on good environment and policy support, but also improve their education level through reading, self-examination, courses, etc., pay attention to the development of internal psychological capital, pay attention to the cultivation of talents and the psychology of employees. Capital development to make knowledge sharing and information transmission more active within the enterprise.

## Conclusion

After analyzing the influence of manufacturing entrepreneurs on the indicator system of digital development of equipment manufacturing industry, the conclusions of this paper are as follows.

(1) Further deepen the research on the development of entrepreneurial psychological capital. At present, domestic research on the development of entrepreneur’s psychological capital has not yet formed a complete and reasonable system of entrepreneurial psychological capital development. On the basis of strengthening theoretical research, find a system and method with localization and regionalization that is more suitable for entrepreneurial psychological capital development, which can enhance regional entrepreneurship and enable enterprises to maintain their advantages in the fierce market competition.

(2) Optimize the business environment and strengthen the propaganda of entrepreneurship. A good business environment enables entrepreneurs to give full play to their own advantages, drives employees to innovate actively, forms a benign environment for cooperation and competition among enterprises, establishes a model of entrepreneurship, and improves support for entrepreneurs. Let entrepreneurs understand that the development of enterprises not only depends on policy support and capital operation, but also needs to pay attention to the cultivation and improvement of their own inner spirit.

## Data availability statement

The original contributions presented in the study are included in the article/supplementary material, further inquiries can be directed to the corresponding author.

## Author contributions

LZ: methodology, data analysis and writing. JL: data analysis and writing-reviewing and editing, article review and intellectual concept of the article. All authors contributed to the article and approved the submitted version.

## Funding

This study has been graciously supported by the National Statistical Science Research Project of China (Grant No. 2022LY094); Philosophy and Social Science Research in Colleges and Universities in Jiangsu Province (Grant No. 2021SJA1926) and University level scientific research project of Yancheng Institute of Technology in 2021 (talents) (Grant No. xjr2021049).

## Conflict of interest

The authors declare that the research was conducted in the absence of any commercial or financial relationships that could be construed as a potential conflict of interest.

## Publisher’s note

All claims expressed in this article are solely those of the authors and do not necessarily represent those of their affiliated organizations, or those of the publisher, the editors and the reviewers. Any product that may be evaluated in this article, or claim that may be made by its manufacturer, is not guaranteed or endorsed by the publisher.
